# Supplementary material for: Muscarinic acetylcholine receptor signaling generates OFF selectivity in a simple visual circuit
Source: Nat Commun. 2019 Sep 9;10:4093. doi: 10.1038/s41467-019-12104-w (PMC6733798; doi:10.1038/s41467-019-12104-w)
Supplement: Supplementary file 1 — Supplementary Information [file 41467_2019_12104_MOESM1_ESM.pdf]

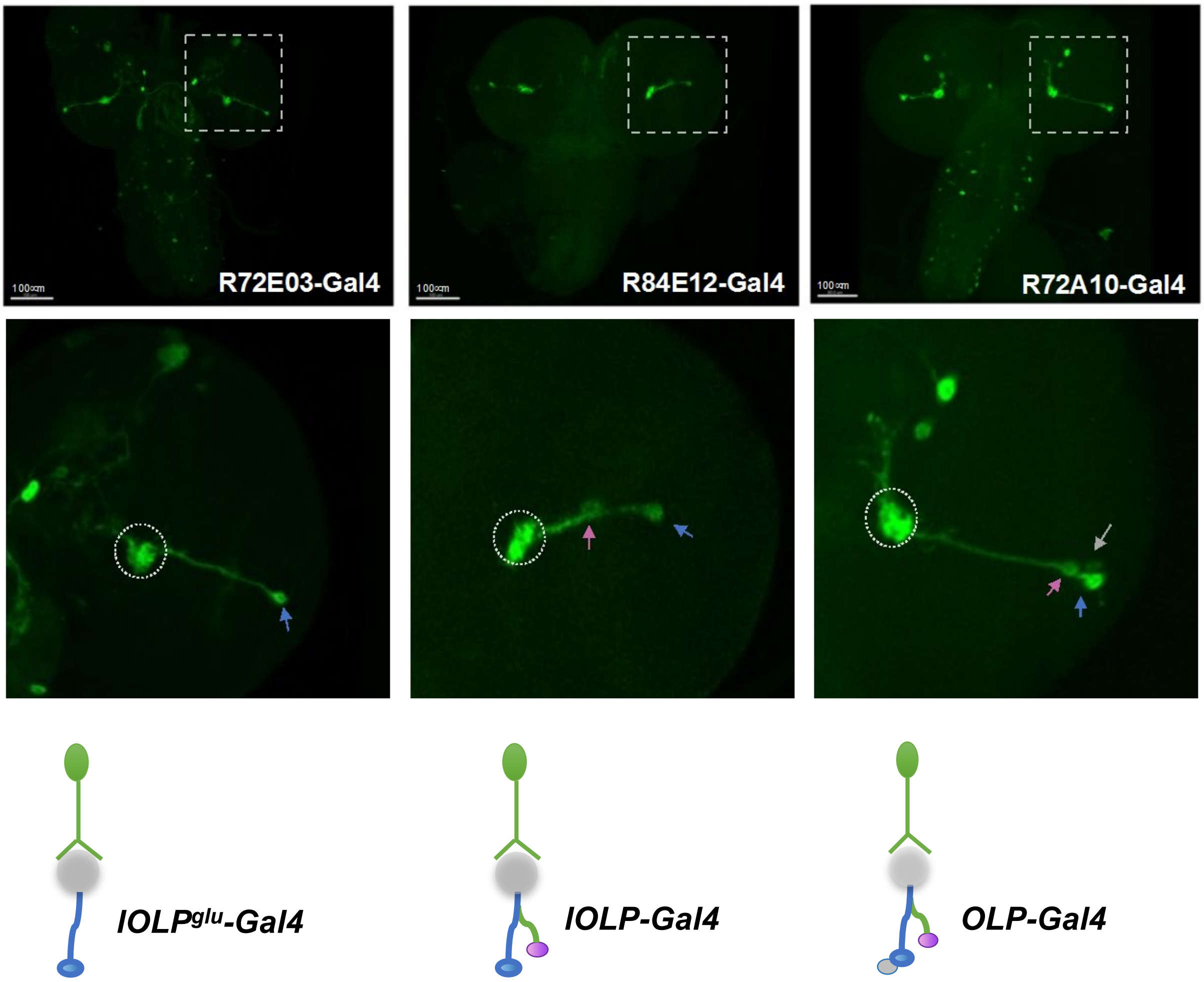

Supplementary Figure 1. Enhancer-Gal4 lines used for labeling and manipulating the OLPs. Three Gal4 lines were selected from the Janelia Farm FlyLight Gal4 collection. Top: Representative projected confocal images showing enhancer Gal4 driven mCD8::GFP expression in larval brain tissues. Middle: zoomed-in views of the OLPs. Grey dashed circles: OLPs' processes in the LON. Pink arrow: cha-lOLP, blue arrow: glu-lOLP, grey arrow: pOLP. Bottom: schematic illustrations showing Gal4s labeling 1, 2 and 3 OLPs. Scale bar = 100 μm. Genotypes are as indicated.

Supplementary Figure 1

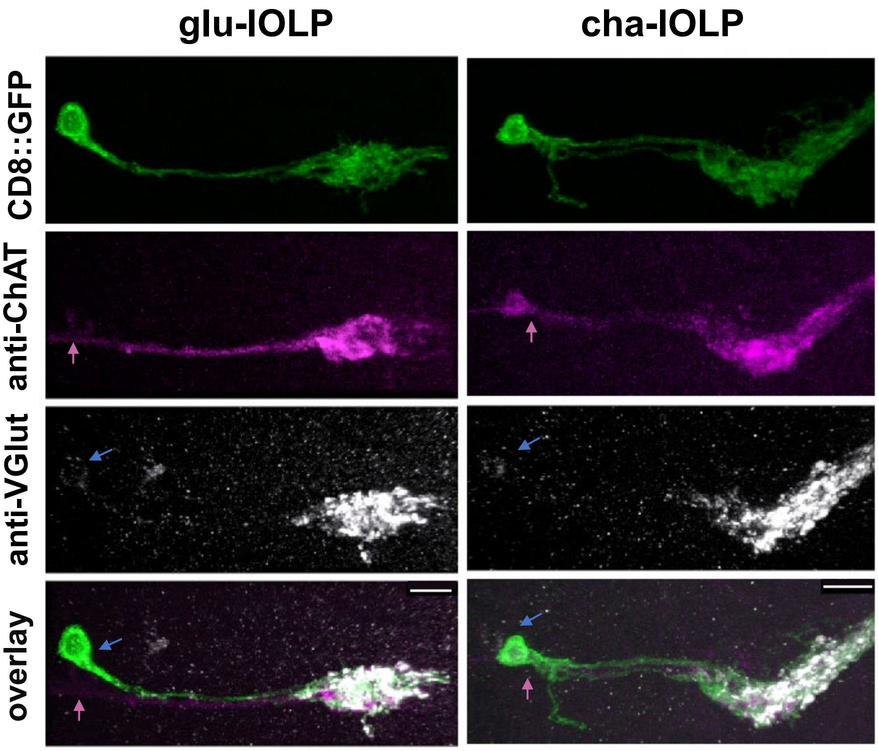

Supplementary Figure 2. **R84E12-Gal4 (lOLP-Gal4) labels glu-lOLP and cha-lOLP.** Projection patterns of the two lOLPs are shown with processes terminating in the LON. The lOLPs were individually labeled by the Flip-out technique (hs-flp; lOLP-Ga4; UAS>CD2>CD8::GFP) and the identities of the lOLPs confirmed by immunostaining with anti-ChAT and anti-VGlut antibodies. Pink arrow: cha-lOLP, blue arrow: glu-lOLP. Scale bar = 20μm.

Supplementary Figure 2

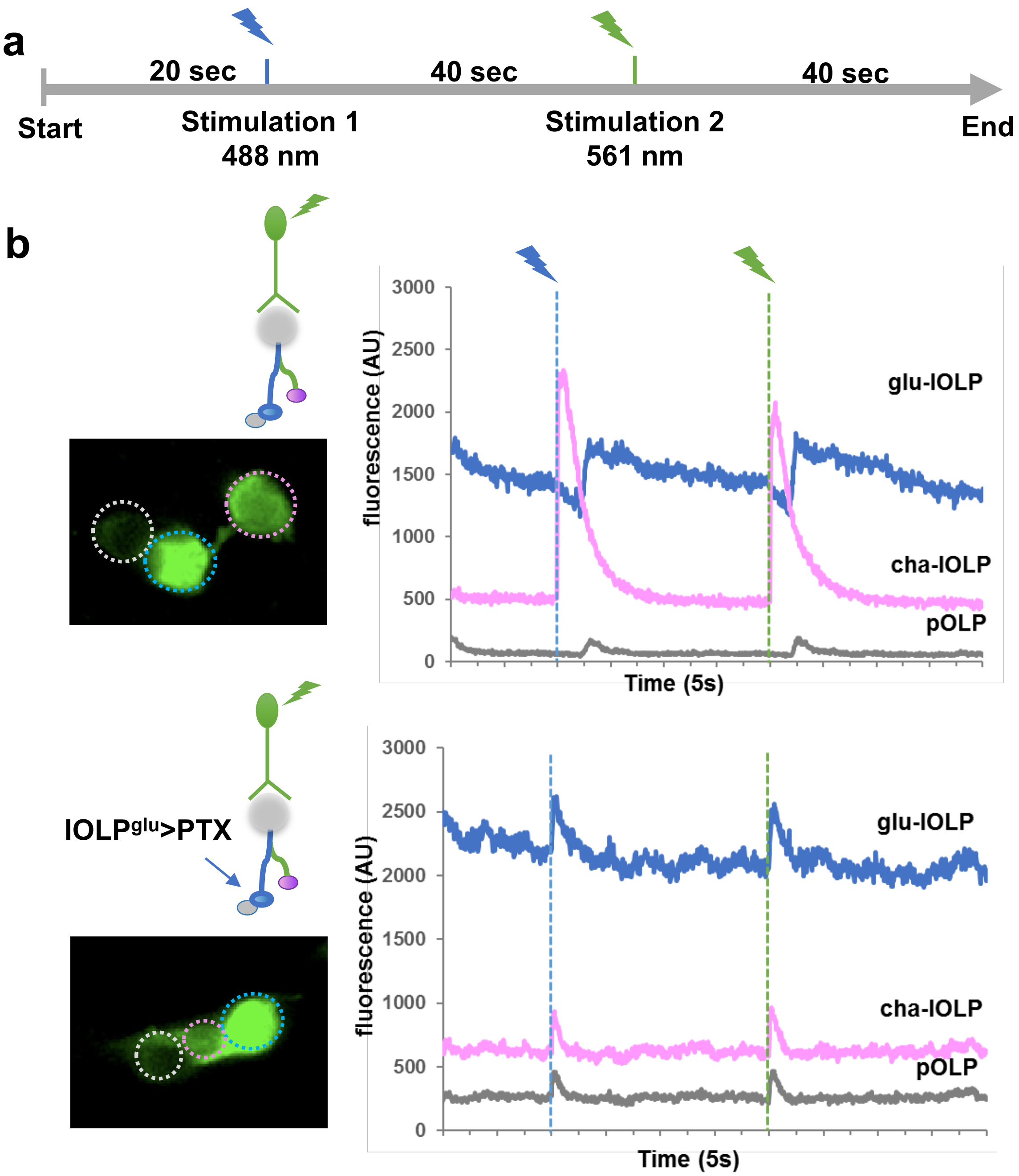

Supplementary Figure 3. glu-lOLP inhibits cha-lOLP and activates pOLP. (a) Schematic illustration of a recording session with two 100 ms light pulses delivered at 488 nm (blue) or 561 nm (green). **(b)** PTX expression in glu-lOLP modifies the temporal profile of its light response and also leads to the dampened response in cha-lOLP and the accelerated response in pOLP. Representative frame and raw traces of the GCaMP recordings are shown. Top: the control with OLP-LexA driven LexAop-GCaMP6f expression. Bottom: UAS-PTX expression driven by lOLP^{glu}-Gal4 was used to specifically modify the temporal profile of glu-lOLP. Somas are marked by dashed circles. Magenta: cha-lOLP, blue: glu-lOLP, grey: pOLP. Dashed lines indicate light pulses.

Supplementary Figure 3

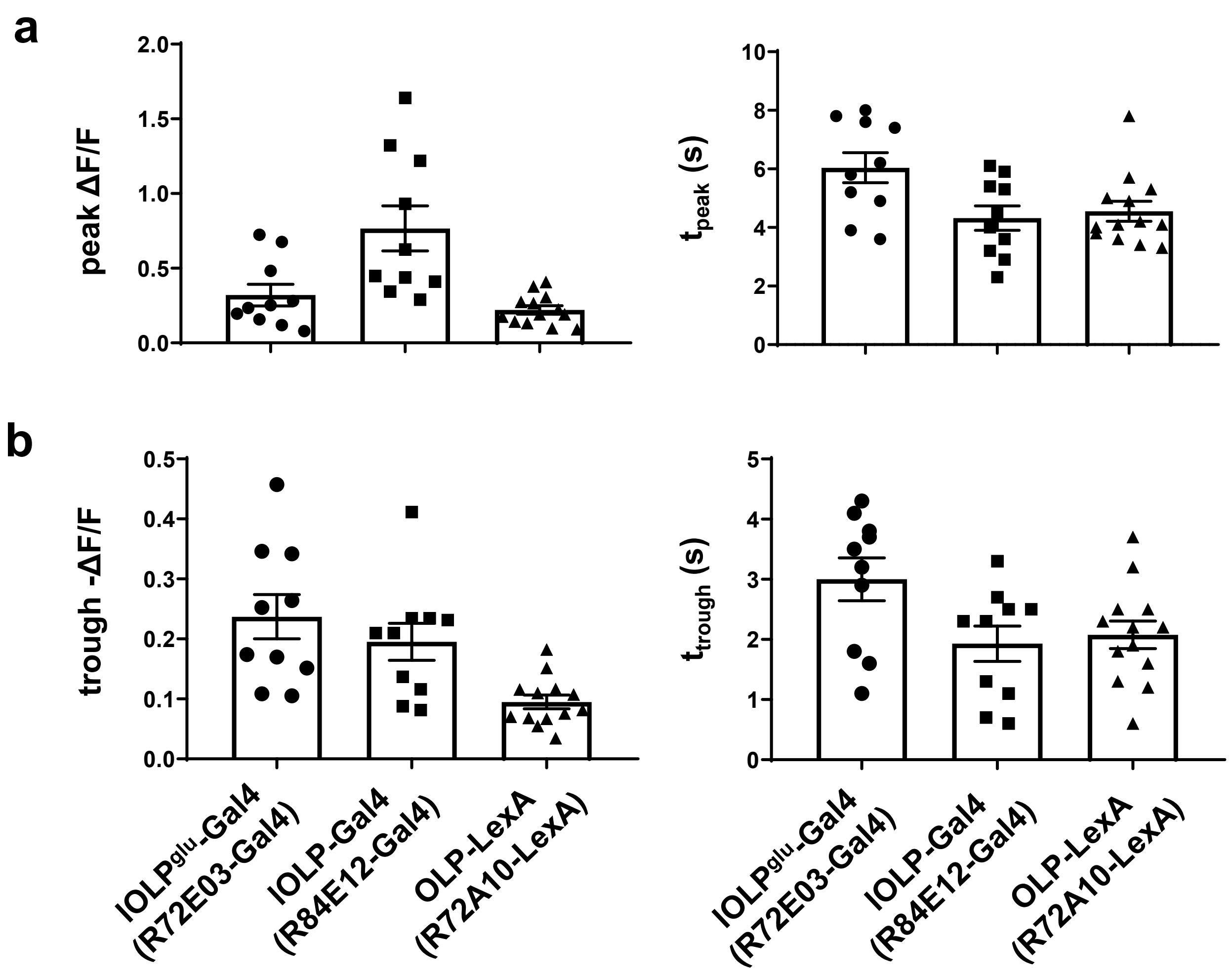

Supplementary Figure 4. Comparison of the glu-lOLP calcium imaging results obtained using three enhancer driver lines. GCaMP6f expression in glu-lOLP driven by different enhancer lines were used to measure light-induced calcium responses, which exhibited a biphasic waveform with a initial reduction followed by a slow rise of the GCaMP signals. The amplitude and latency of the peak and trough were quantified and the results obtained from the three drivers are comparable. Genotypes are as indicated. Quantifications of **(a)** peak value and peak time of changed intensity (ΔF/F) are shown as well as **(b)** trough value and trough time of changed intensity (-ΔF/F). n = 10, lOLP^{glu}-Gal4; n = 10, lOLP-Gal4; n = 13 OLP-LexA.

Supplementary Figure 4

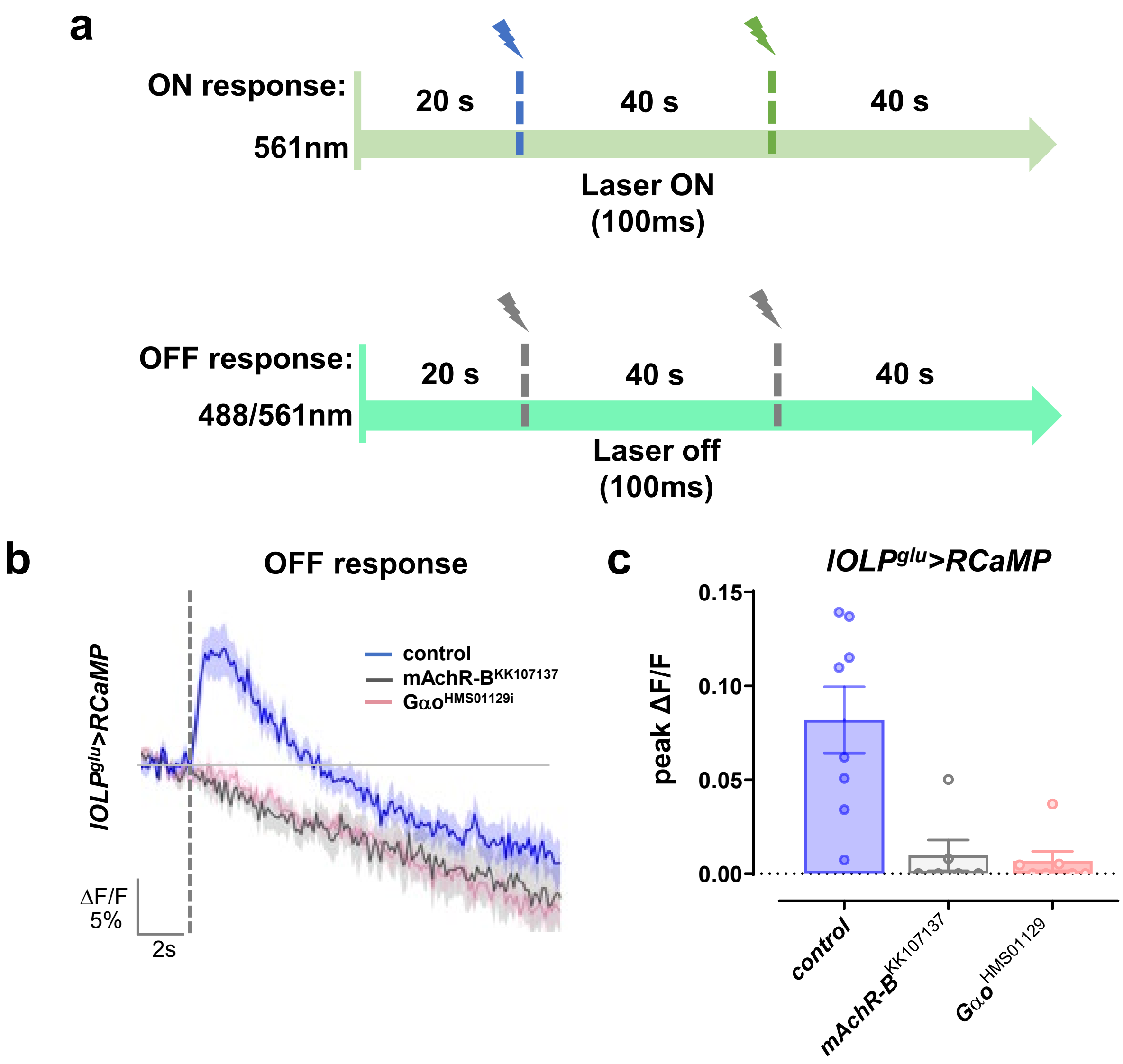

Supplementary Figure 5. The OFF response in glu-IOLP is mediated by the mAchR-B receptor and Gαo signaling. (**a**) Schematic illustration of recordings examining the ON and OFF responses using IOLP-Gal4 driving RCaMP. The ON response was recorded using a 561 nm confocal laser at low intensity and stimulated with the confocal laser at high intensity for 100 ms. The OFF response was recorded by turning off the lasers for 100 ms. The stimulations are contrast matched. Please see Methods for details. (**b**) Dark pulse-induced OFF responses were eliminated in the glu-IOLP by RNAi knock-downs of mAchR-B and Gαo. Average IOLP^{glu}-Gal4>RCaMP traces are shown. The grey dashed line indicates the 100 ms dark pulse. The genotypes are as indicated. n = 7 in all groups. **(c)** RNAi knockdown of mAchR-B or Gαo demonstrates significant reductions in the amplitude of the calcium responses. Quantification of changed intensity (ΔF/F) is shown. Control, n = 8; mAchR-B^{RNAi}, n = 6; Gαo^{RNAi}, n = 7.

Supplementary Figure 5

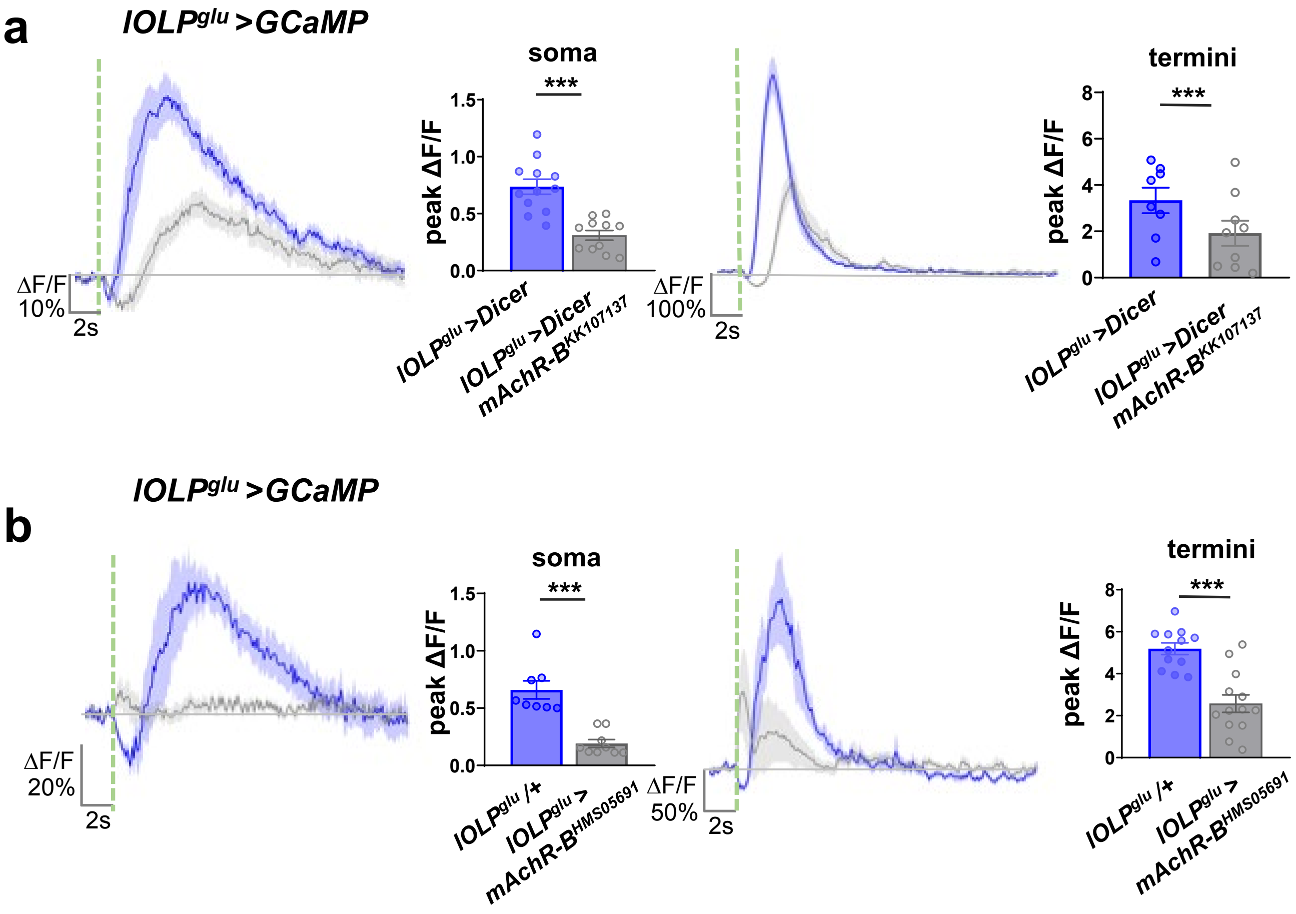

Supplementary Figure 6. Knocking-down of mAchR-B consistently dampens the light-induced calcium responses in glu-IOLP. GCaMP signals were recorded in the soma (left) and termini (right) of glu-IOLPs, both of which showed significant decreases in amplitudes with the mAchR-B knock-down. Two transgenic RNAi lines, **(a)** mAchR-$B^{KK107137}$ and **(b)** mAchR-$B^{HMS05691}$, generated responses with different kinetics. Average traces and the quantification of peak changed intensity (ΔF/F) is shown. The green dashed line indicates the 100 ms light pulse. $IOLP^{glu}$>Dicer, n = 12; $IOLP^{glu}$>Dicer, mAchR-$B^{KK107137}$ = 11; $IOLP^{glu}$/+, n = 8; $IOLP^{glu}$>mAchR-$B^{HMS05691}$, n = 9.

Supplementary Figure 6

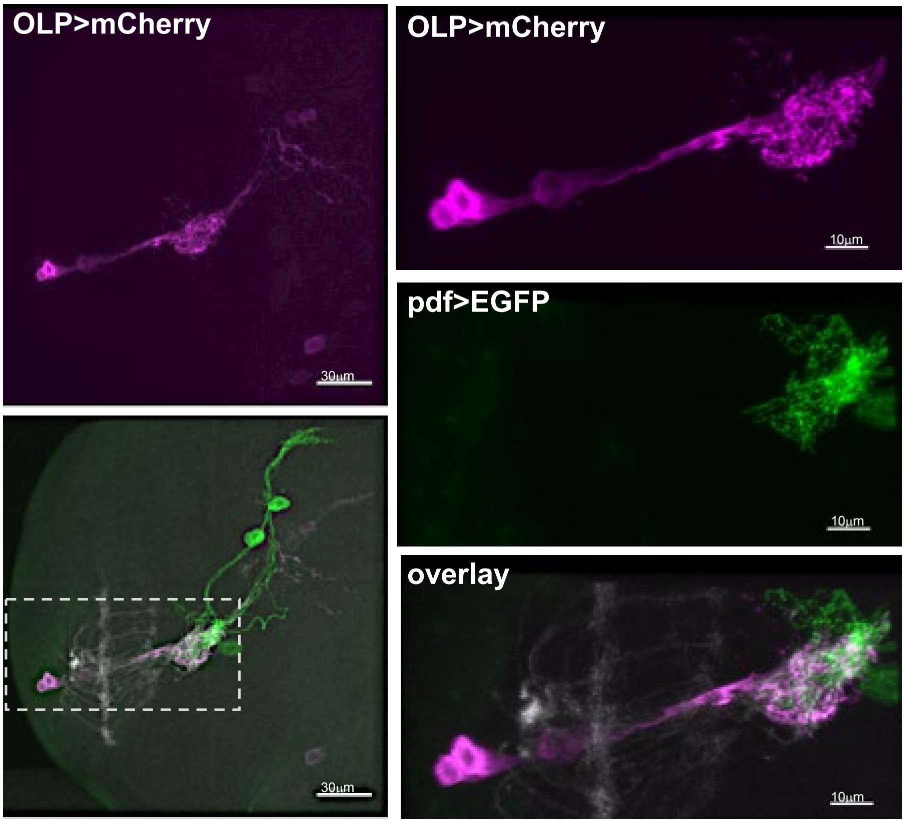

Supplementary Figure 7. LNvs are visual projection neurons receiving synaptic input from PRs and OLPs. Representative confocal images illustrating the anatomical connections among PR axon terminals stained by 24B10 antibody (grey), the projections of OLPs (OLP-LexA>LexAop-mCherry, magenta) and dendritic arbors of LNvs (pdf>CD8::GFP, green) in the larval LON.

Supplementary Figure 7

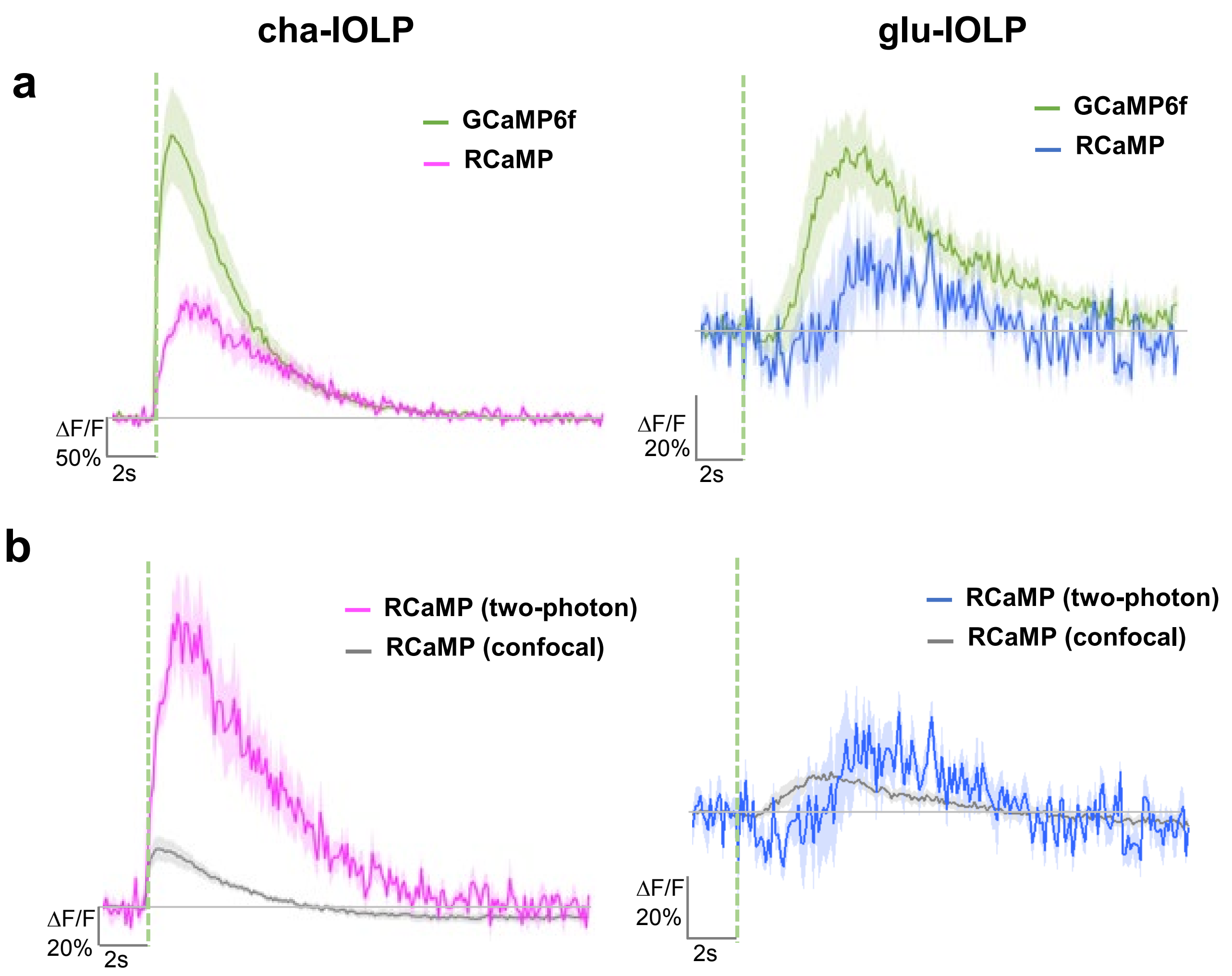

Supplementary Figure 8. Comparison between calcium imaging results obtained using RCaMP vs. GCaMP6f and confocal vs. two-photon. (a) Calcium responses of the cha-lOLP (pink) and glu-lOLP (blue) were recorded using RCaMP or GCaMP6f with a two-photon laser tuned to 1040 nm or 920 nm, respectively. Although waveforms of the calcium transients are similar, the GCaMP recordings (green traces) showed responses with higher amplitude compared to the RCaMP recordings. **(b)** RCaMP recordings imaged using a two-photon laser tuned to 1040 nm produced calcium transients with higher amplitudes than the ones imaged using a confocal laser tuned to 561 nm (grey traces). The average traces are shown. Shaded areas represent SEM. 100 ms light pulses were delivered using the 561 nm laser and are indicated by the dashed lines.

Supplementary Figure 8
