# Supplementary material for: Muscarinic acetylcholine receptor signaling generates OFF selectivity in a simple visual circuit
Source: Nat Commun. 2019 Sep 9;10:4093. doi: 10.1038/s41467-019-12104-w (PMC6733798; doi:10.1038/s41467-019-12104-w)
Supplement: Supplementary file 3 — Reporting Summary [file 41467_2019_12104_MOESM3_ESM.pdf]

nature research

Corresponding author(s): Quan Yuan

Last updated by author(s): Aug 15, 2019

Reporting Summary

Nature Research wishes to improve the reproducibility of the work that we publish. This form provides structure for consistency and transparency in reporting. For further information on Nature Research policies, see Authors & Referees and the Editorial Policy Checklist.

Statistics

For all statistical analyses, confirm that the following items are present in the figure legend, table legend, main text, or Methods section.

n/a	Confirmed	
☐	☒	The exact sample size (*n*) for each experimental group/condition, given as a discrete number and unit of measurement
☐	☒	A statement on whether measurements were taken from distinct samples or whether the same sample was measured repeatedly
☐	☒	The statistical test(s) used AND whether they are one- or two-sided<br>*Only common tests should be described solely by name; describe more complex techniques in the Methods section.*
☐	☒	A description of all covariates tested
☐	☒	A description of any assumptions or corrections, such as tests of normality and adjustment for multiple comparisons
☐	☒	A full description of the statistical parameters including central tendency (e.g. means) or other basic estimates (e.g. regression coefficient) AND variation (e.g. standard deviation) or associated estimates of uncertainty (e.g. confidence intervals)
☐	☒	For null hypothesis testing, the test statistic (e.g. *F*, *t*, *r*) with confidence intervals, effect sizes, degrees of freedom and *P* value noted<br>*Give P values as exact values whenever suitable.*
☒	☐	For Bayesian analysis, information on the choice of priors and Markov chain Monte Carlo settings
☒	☐	For hierarchical and complex designs, identification of the appropriate level for tests and full reporting of outcomes
☒	☐	Estimates of effect sizes (e.g. Cohen's *d*, Pearson's *r*), indicating how they were calculated

Our web collection on statistics for biologists contains articles on many of the points above.

Software and code

Policy information about availability of computer code

Data collection	Image data collection: Zen-black, 2011, Zeiss, Germany.
Data analysis	Image data analysis: custom written MATLAB scripts. Available upon request.

For manuscripts utilizing custom algorithms or software that are central to the research but not yet described in published literature, software must be made available to editors/reviewers. We strongly encourage code deposition in a community repository (e.g. GitHub). See the Nature Research guidelines for submitting code & software for further information.

Data

Policy information about availability of data

All manuscripts must include a data availability statement. This statement should provide the following information, where applicable:

- Accession codes, unique identifiers, or web links for publicly available datasets
- A list of figures that have associated raw data
- A description of any restrictions on data availability

All data supporting the findings in this study are available from the corresponding author upon reasonable request. The source data underlying Figs 1d-e, 2d, 3a, b, d, 4e, 5a-d, f, 6a, d, 7c, 8c, e, and Supplementary Figs 4a, b, 5c and 6a, b are provided as a Source Data file.

Field-specific reporting

Please select the one below that is the best fit for your research. If you are not sure, read the appropriate sections before making your selection.

☒ Life sciences ☐ Behavioural & social sciences ☐ Ecological, evolutionary & environmental sciences

Life sciences study design

All studies must disclose on these points even when the disclosure is negative.

Sample size	For functional imaging experiments, we collected 6 to 15 samples for each genotype and condition. This number is in accordance with similar studies in the field and is limited by the number of animals with the desired genotype.
Data exclusions	No data were exclueded.
Replication	We performed genetic analyses using different genetic reagents to ensure the reproducibility of the experimental findings. Experiments were performed and repeated by four different lab members. All findings can be reproduced.
Randomization	The sample collection was not randomized due to the large number of genotypes we tested. The data analyses and graphing were performed using a custom written MATLAB script to reduce human biases.
Blinding	The data analyses were performed by MATLAB scripts blindly with fixed parameters for all groups.

Reporting for specific materials, systems and methods

We require information from authors about some types of materials, experimental systems and methods used in many studies. Here, indicate whether each material, system or method listed is relevant to your study. If you are not sure if a list item applies to your research, read the appropriate section before selecting a response.

Materials & experimental systems

n/a	Involved in the study	
☐	☒	Antibodies
☒	☐	Eukaryotic cell lines
☒	☐	Palaeontology
☐	☒	Animals and other organisms
☒	☐	Human research participants
☒	☐	Clinical data

Methods

n/a	Involved in the study	
☒	☐	ChIP-seq
☒	☐	Flow cytometry
☒	☐	MRI-based neuroimaging

Antibodies

Antibodies used	Primary antibodies used were rabbit anti-GFP antibody (Abcam, Ab6556, 1: 200), mouse anti-ChAT (DSHB, ChAT4B1, 1:10), and rabbit anti-VGluT (a gift from Dr. DiAntonio, 1:5000). Secondary antibodies used were goat anti-rabbit Alex 633 (Invitrogen, A-21070) and donkey anti-mouse CY3 (Jackson ImmunoResearch Labs, 715165150).
Validation	Citations for anti-ChAT and anti-VGluT antibodies are included in the manuscript, Reference# 24to 26. Validation statements for the commercial antibodies are available on the manufactures websites.

Animals and other organisms

Policy information about studies involving animals; ARRIVE guidelines recommended for reporting animal research

Laboratory animals	Drosophila melanogaster is used in the study. Both male and female are collected at the wandering third instar larval stage for the live imaging study and behavior analyses. The genotypes are described in the main text and Methods. No animal protocol is required for studies using Drosophila.
Wild animals	none
Field-collected samples	none
Ethics oversight	none

Note that full information on the approval of the study protocol must also be provided in the manuscript.
